# Decreased Alu methylation in type 2 diabetes mellitus patients increases HbA1c levels

**DOI:** 10.1002/jcla.24966

**Published:** 2023-09-24

**Authors:** Jirapan Thongsroy, Apiwat Mutirangura

**Affiliations:** ^1^ School of Medicine Walailak University Nakhon Si Thammarat Thailand; ^2^ Research Center in Tropical Pathobiology Walailak University Nakhon Si Thammarat Thailand; ^3^ Center for Excellence in Molecular Genetics of Cancer and Human Diseases Chulalongkorn University Bangkok Thailand; ^4^ Department of Anatomy, Faculty of Medicine Chulalongkorn University Bangkok Thailand

**Keywords:** aging, Alu, DNA methylation, genomic instability, type 2 DM

## Abstract

**Introduction:**

Alu hypomethylation is a common epigenetic process that promotes genomic instability with aging phenotypes, which leads to type 2 diabetes mellitus (type 2 DM). Previously, our results showed significantly decreased Alu methylation levels in type 2 DM patients. In this study, we aimed to investigate the longitudinal changes in Alu methylation levels in these patients.

**Results:**

We observed significantly decreased Alu methylation levels in type 2 DM patients compared with normal (*p* = 0.0462). Moreover, our findings demonstrated changes in Alu hypomethylation over a follow‐up period within the same individuals (*p* < 0.0001). A reduction in Alu methylation was found in patients with increasing HbA1c levels (*p* = 0.0013) and directly correlated with increased HbA1c levels in type 2 DM patients (*r* = −0.2273, *p* = 0.0387).

**Conclusions:**

Alu methylation in type 2 DM patients progressively decreases with increasing HbA1c levels. This observation suggests a potential association between Alu hypomethylation and the underlying molecular mechanisms of elevated blood glucose. Furthermore, monitoring Alu methylation levels may serve as a valuable biomarker for assessing the clinical outcomes of type 2 DM.

## Graphical abstract

The flowchart provides a concise presentation of the conceptual framework of our findings. It not only reveals significant Alu hypomethylation in individuals with type 2 DM but also demonstrates a decrease in Alu hypomethylation levels during the follow‐up of type 2 DM patients belonging to the group with increased HbA1c levels. Consequently, Alu methylation holds promise as a potential biomarker for the diagnosis or monitoring of type 2 DM in the future.

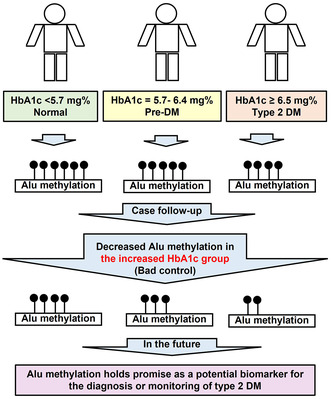

## 1 INTRODUCTION

Type 2 diabetes mellitus (type 2 DM) has been a public health problem for several decades. Currently, an estimated 537 million people are at an increased risk of type 2 DM, and this number is expected to increase to 592 million by 2035 and reach 783 million by 2045.^1^, ^2^ Type 2 DM patients can develop various complications, including cardiovascular disease, stroke, foot ulcers, chronic kidney failure, and diabetic retinopathy,^3^, ^4^, ^5^, ^6^, ^7^, ^8^, ^9^, ^10^ making it one of the world's most critical health concerns.^11^, ^12^ Type 2 DM is a multifactorial condition characterized by high blood glucose levels (hyperglycemia),^13^, ^14^, ^15^ which are influenced by genetic and epigenetic factors.^16^, ^17^, ^18^, ^19^ DNA methylation, an epigenetic event commonly found in elderly individuals, exhibits changes in type 2 DM patients and correlates with poor health status in humans.^20^, ^21^, ^22^, ^23^, ^24^, ^25^, ^26^, ^27^

DNA methylation serves two fundamental purposes: genomic stability prevention and gene expression regulation.^28^, ^29^ Alu methylation, a specific form of DNA methylation, has been shown to play a role in minimizing the accumulation of endogenous DNA damage.^30^ This effect is achieved by reducing the torsional force on the DNA double helix through naturally occurring hypermethylated DNA gaps.^31^, ^32^, ^33^, ^34^, ^35^ Previous studies have indicated that type 2 DM patients accumulated DNA damage,^36^, ^37^, ^38^ leading to genomic instability and cellular senescence.^39^, ^40^ Therefore, it is plausible to consider that Alu hypomethylation‐induced DNA damage is one of the processes contributing to genomic instability in type 2 DM.

Presently, blood tests are used to diagnose type 2 DM by measuring fasting blood sugar (FBS) and hemoglobin A1C (HbA1c) levels,^41^, ^42^, ^43^ and guidelines gauge good diabetes care primarily based on HbA1c levels.^44^, ^45^ Although it is an effective diagnostic test for type 2 DM,^46^ it is mainly used to detect the disease in patients who already exhibit symptoms. Therefore, blood test results for type 2 DM diagnosis should be confirmed with other measurements before patients display DM symptoms, such as Alu methylation levels.

In this study, we employed ALU‐Combined Bisulfite Restriction Analysis (COBRA) to assess Alu methylation in all samples (Figure S1). COBRA, a highly precise quantitative method for measuring methylation, was used as it provides more detailed information about DNA methylation patterns compared to pyrosequencing.^47^, ^48^, ^49^ In our previous study, we categorized the Alu methylation pattern in our samples, which included control, pre‐DM, and type 2 DM patients. Our findings revealed lower Alu methylation levels in type 2 DM patients compared to the general population.^50^ Here, we observed Alu methylation levels during 4 years of follow‐up in normal, pre‐DM, and type 2 DM groups.

We hypothesized that the extent of Alu hypomethylation would undergo changes within the same individuals over the follow‐up period. The aim of this study was to determine whether Alu methylation could serve as a highly specific novel biomarker for more accurate screening and monitoring of the progression of type 2 DM in the future.

## 2 MATERIALS AND METHODS

### 2.1 Participants

We conducted this study with a total of 203 samples. The participants' blood glucose levels were monitored using HbA1c, and they were divided into three groups: normal (56 samples), pre‐DM (64 samples), and type 2 DM (83 samples). The pre‐DM and type 2 DM patients were admitted to Thailand's Tambon Health Promoting Hospital. The age range of the patients was 31 to 85 years. The participants were recruited for the study and provided written informed consent. Access to participant information that could identify individuals was maintained during data collection. All methods were performed in accordance with the relevant guidelines and regulations. Approval for the study was obtained on September 28, 2015, from the Ethics Clearance Committee on Human Rights Related to Research Involving Human Subjects at Walailak University in Nakorn Sri Thammarat, Thailand, in accordance with the Declaration of Helsinki. The study has been reviewed and approved by the Thai Clinical Trials Registry Committee with the identification number TCTR20220324001.

### 2.2 DNA extraction and bisulfite DNA modification

DNA was extracted from the buffy coat using proteinase K digestion and phenol–chloroform extraction protocols. The denatured genomic DNA was then incubated at 37°C for 10 min in 0.22 M NaOH, followed by incubation with 30 μL of 10 mM hydroquinone and 520 μL of 3 M sodium‐bisulfite at 50°C for 16–20 h. Subsequently, the DNA was purified and treated with 0.33 M NaOH for 3 min at 25°C, after which it was ethanol precipitated, washed with 70% ethanol, and finally resuspended in 20 μL of H_2_O.^50^

### 2.3 ALU‐Combined bisulfite restriction analysis (COBRA)

To detect methylated levels at thousands of CpG loci, a set of conserved primers for each IRS (interspersed repetitive sequence) was used. The following components were used to determine the methylation level of Alu in the samples: 1X PCR buffer (Qiagen, Germany), 0.2 mM deoxynucleotide triphosphate (dNTP) (Promega, USA), 1 mM magnesium chloride (Qiagen, Germany), 25 U of HotStarTaq DNA Polymerase (Qiagen, Germany), and a primer pair at a concentration of 0.3 μM. The primer sequences used for Alu amplification were as follows: ALU‐Forward (5′‐GGYGYGGTGGTTTAYGTTTGTAA‐3′) and ALU‐Reverse (5′‐CTAACTTTTTATATTTTTAATAAAAACRAAATTTCACCA‐3′), where R represents A and G, and Y represents C and T. The PCR program for Alu amplification consisted of an initial denaturation step at 95°C for 15 min, followed by 40 cycles of denaturation at 95°C for 45 s, annealing at 57°C for 45 s, extension at 72°C for 45 s, and a final extension step at 72°C for 7 min. After the Alu PCR products were obtained, COBRA was performed. The COBRA reaction included 2 U of TaqI (Thermo Scientific, USA), 2 U of TasI (Thermo Scientific, USA), 5X NEB3 buffer (New England Biolabs, USA), and 1 μg/μL bovine serum albumin (BSA) (New England Biolabs, USA). Each digestion reaction was incubated overnight at 65°C, followed by separation on an 8% acrylamide gel and staining with SYBR (Lonza, USA). The intensity of the Alu methylation band was observed and measured using a phosphoimager and ImageQuant software (GE Healthcare, UK).^50^

### 2.4 Methylation analysis

The COBRA results were categorized into the following four groups based on the methylation status of the two CpG dinucleotides: hypermethylation at both CpGs (^m^C^m^C); hypomethylation at CpGs (^u^C^u^C); partial methylation of the ^m^C^u^C; and partial methylation of the ^u^C^m^C. To calculate the Alu methylation levels for each group, the intensity of COBRA‐digested Alu products was measured using a Typhoon FLA 7000 biomolecular imager (GE Healthcare, UK). The band intensities of five Alu products with sizes of 133, 90, 75, 58, and 43 bp were used in the Alu methylation analysis. The following formula was employed: A = 133/133, B = 58/58, C = 75/75, D = 90/90, E = 43/43, and F = 32/32. The Alu methylation levels were calculated using the following formulas: Alu methylation level percentage (%^m^C) = 100 × (E + B)/(2A + E + B + C + D); percentage of ^m^C^m^C loci (%^m^C^m^C) = 100 × F/(A + C + D + F); percentage of ^u^C^m^C loci (%^u^C^m^C) = 100 × C/(A + C + D + F); percentage of ^m^C^u^C loci (%^m^C^u^C) = 100 × D/(A + C + D + F); and percentage of ^u^C^u^C loci (%^u^C^u^C) = 100 × A/(A+ C + D + F). These formulas allow for the calculation of Alu methylation levels based on the band intensities obtained from the COBRA analysis.

### 2.5 Statistical analyses

The average and distributions of characteristic data for all the samples were analyzed and presented as the mean ± SD and median. Differences in blood glucose levels between groups in matched cases were determined using *t*‐tests, with a significance threshold set at a *p* value of 0.05. To explore the relationship between two continuous variables, Spearman's correlation coefficient was utilized.

## 3 RESULTS

### 3.1 Alu methylation levels in patients with type 2 DM

**FIGURE 1:**
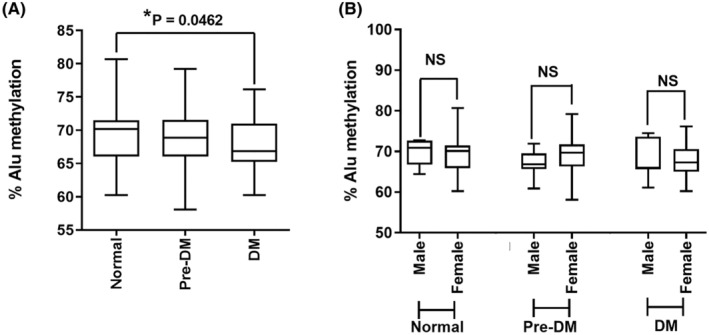
The Alu methylation levels in the samples were analyzed based on the HbA1c levels. (A) The percentage of each Alu methylation level in normal, pre‐DM, and type 2 DM samples. (B) The Alu methylation levels in males and females. The results are presented as box plots, where the boxes represent the interquartile ranges (25th to 75th percentile), the median lines represent the 50th percentile, and the whiskers represent the minimum and maximum values *p < 0.05 (*t* test) (Mann–Whitney test).

In this study, a total of 203 samples were categorized into three groups based on their HbA1c levels; there were 56 normal control subjects, 64 pre‐DM patients, and 83 type 2 DM patients (Table 1). The levels of Alu methylation in the normal group were compared to those in the pre‐DM and type 2 DM groups. Our analysis revealed that type 2 DM patients exhibited significantly lower Alu methylation levels than normal control subjects (*p* = 0.0462) (Figure 1A). To investigate the potential influence of sex differences on Alu methylation levels, we further examined and compared the methylation levels between males and females within each group. Our findings indicated that there were no significant differences in Alu methylation levels between males and females across all three groups (Figure 1B).

**TABLE 1:** Sample size, sex, age, and body mass index (BMI) in each group by HbA1c indicator.

HbA1c indicator	Group			*p* Value
	Normal	Pre‐DM	Type 2 DM	
*N*	56	64	83	
Sex				
Male	7 (12.50%)	13 (20.31%)	17 (20.48%)	
Female	49 (87.50%)	51 (79.69%)	66 (79.52%)	
Age (years) (mean ± SD)	56.05 ± 10.58	54.63 ± 9.78	58.67 ± 11.28	0.0718
BMI (kg/m^2^) (mean ± SD)	24.80 ± 3.48	25.25 ± 3.74	25.66 ± 4.39	0.4843

### 3.2 Alu methylation levels during case follow‐up

**FIGURE 2:**
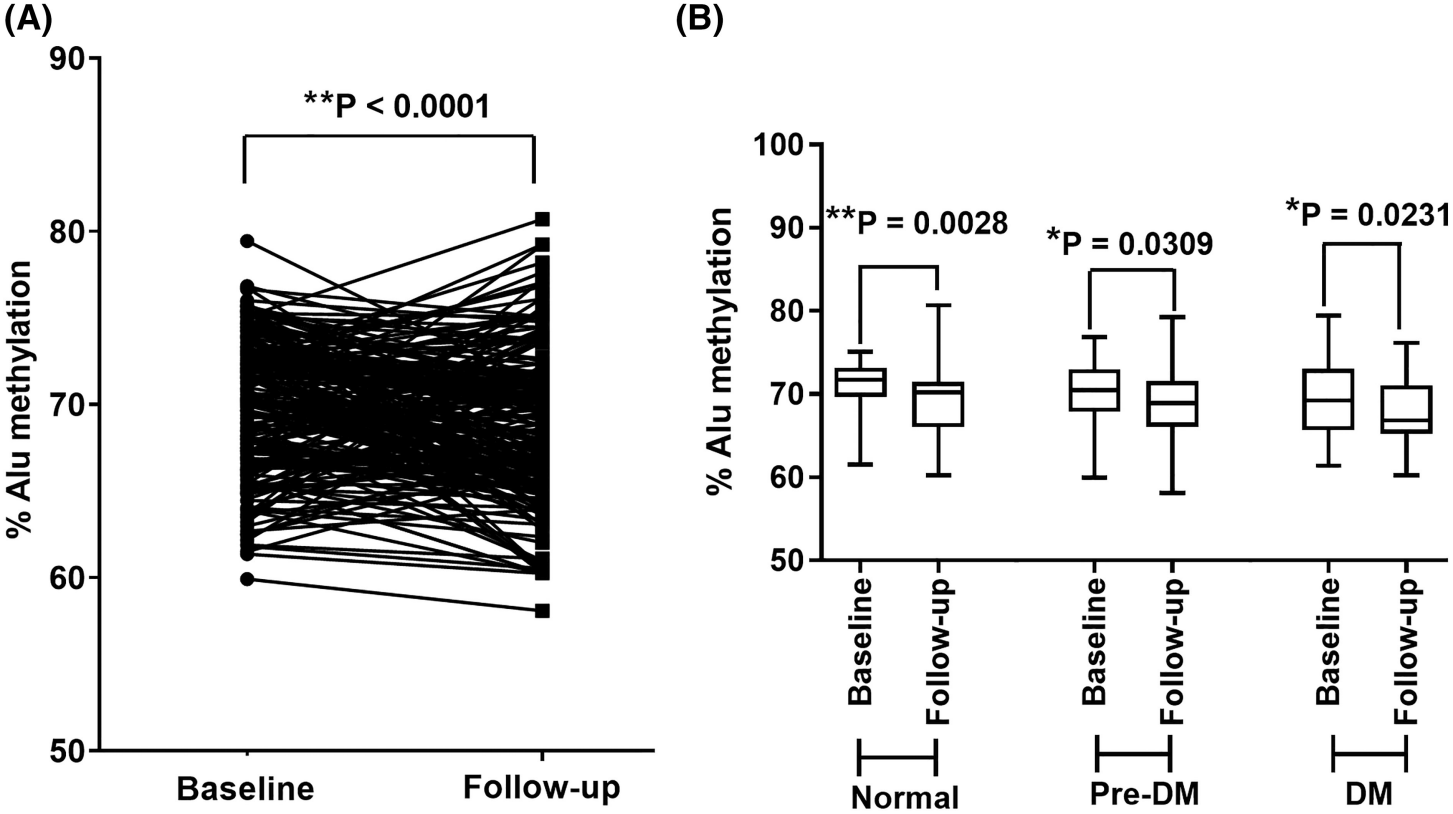
Alu methylation levels over a follow‐up period in the same individuals from all the samples (A). (B) The percentage distribution of each follow‐up level in the normal, pre‐DM, and type 2 DM samples. *p < 0.05, **p < 0.001 (Wilcoxon signed‐rank test).

The results also showed significantly different levels of Alu methylation for 4 years of follow‐up in all the samples (Figure 2A) (*p* < 0.0001). Additionally, when we classified the samples based on the HbA1c indicator, the Alu methylation levels in the normal, pre‐DM, and type 2 DM groups were significantly decreased (Figure 2B) (*p* = 0.0028, *p* = 0.0309, and *p* = 0.0231, respectively).

**FIGURE 3:**
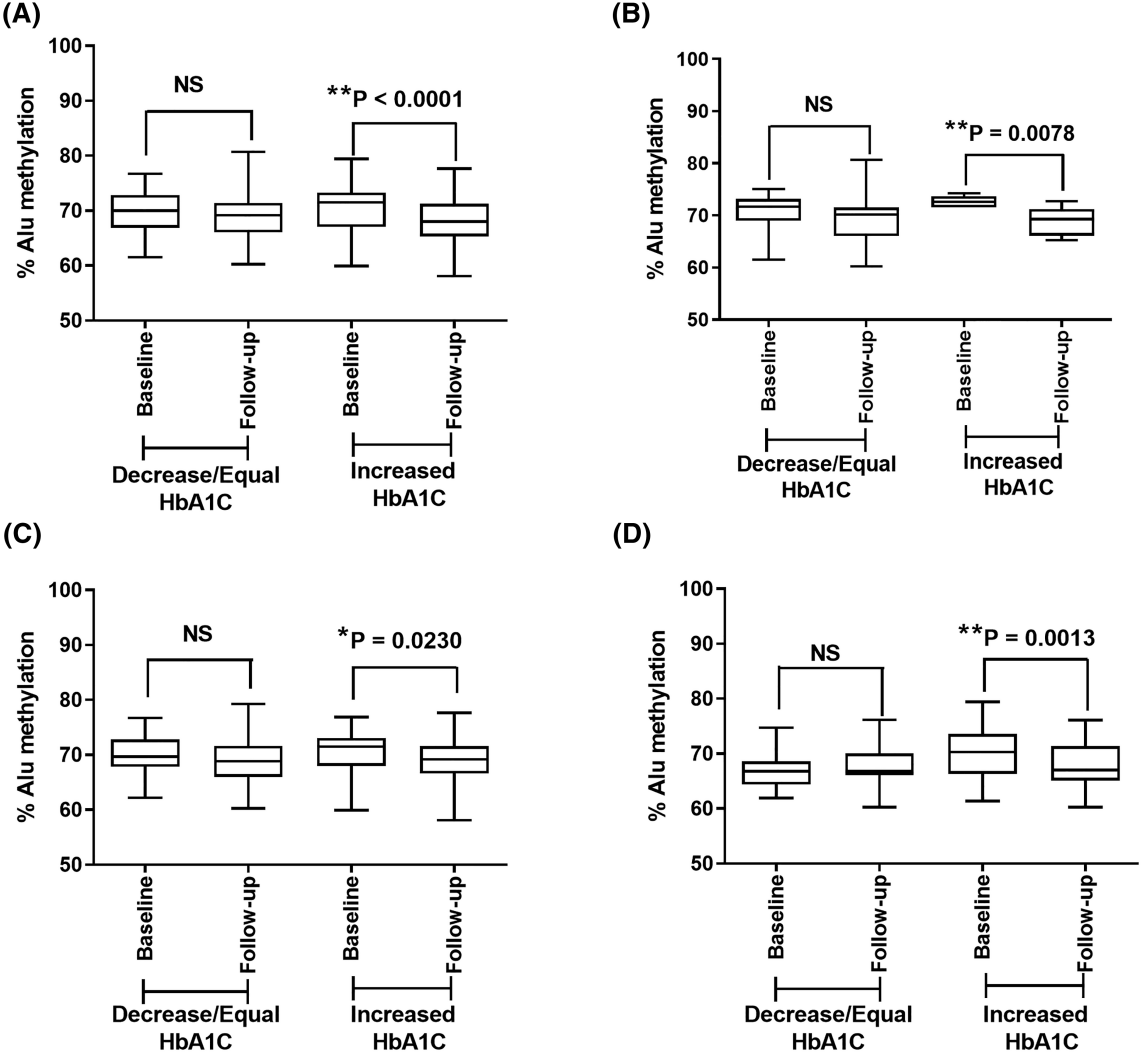
Alu methylation levels of follow‐up samples with a cutoff point determined by the change in HbA1c levels in all samples (A), normal (B), pre‐DM (C), and type 2 DM groups (D). *p < 0.05, **p < 0.001 (Wilcoxon signed‐rank test).

Furthermore, we further divided the three groups (normal, pre‐DM, and type 2 DM) into two subgroups based on changes in HbA1c levels during the case follow‐up. The first subgroup represented individuals with decreased or equal HbA1c levels, and the second subgroup included those with increased HbA1c levels. Our results demonstrated a significant decrease in Alu methylation levels in the subgroup with increased HbA1c levels during the case follow‐up, encompassing all the samples, normal, pre‐DM, and type 2 DM groups (Figure 3A–D) (*p* < 0.0001, *p* = 0.0078, *p* = 0.0230, and *p* = 0.0013, respectively).

### 3.3 Correlation between altered Alu methylation and altered HbA1c levels in normal, pre‐DM, and type 2 DM

**FIGURE 4:**
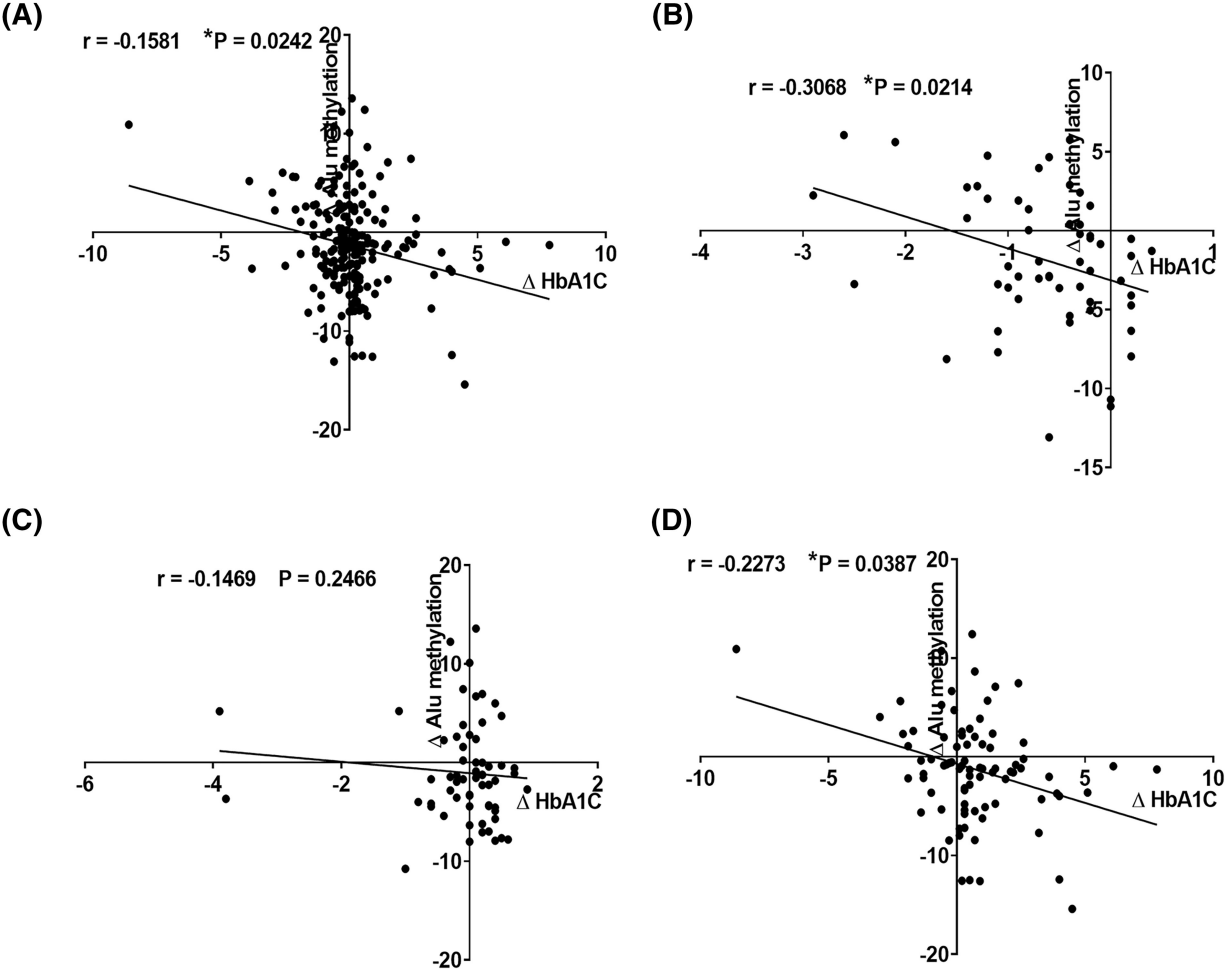
Correlation between ΔAlu methylation levels and ΔHbA1c for all samples (A) and the normal (B), pre‐DM (C), and type 2 DM groups (D). Spearman's correlation coefficients (*r*) with *p* values are indicated (*p < 0.05).

We observed the association between different levels of Alu methylation (ΔAlu methylation) and different levels of HbA1c (ΔHbA1c) in the four‐year follow‐up in all samples, normal, pre‐DM, and type 2 DM patients (Figure 4A–D, respectively). The results showed a significant inverse correlation between ΔAlu methylation and ΔHbA1c in all samples and those from the normal and type 2 DM patients (Figure 4A,B,D) (*r* = −0.1581, *p* = 0.0242, *r* = −0.3068, *p* = 0.0214 and *r* = −0.2273, *p* = 0.0387, respectively).

## 4 DISCUSSION

Previously, our data showed that Alu methylation in patients with type 2 DM was lower than that in the general population. Furthermore, the results demonstrated a direct correlation between Alu hypomethylation levels and high blood glucose in type 2 DM.^50^ Moreover, this association is similar to the correlation between Alu hypomethylation and lower bone mass or hypertension patients.^27^, ^51^ Therefore, the aim of this study was to determine the correlation between changes in Alu methylation levels in individual patients, particularly those who had increased HbA1c levels, and the progression of type 2 DM. Interestingly, our results revealed that Alu methylation levels were consistently reduced in the same individuals, with significantly decreased levels observed in the normal, pre‐DM, and type 2 DM groups during the follow‐up. Alu hypomethylation might represent an epigenetic alteration that plays a key role in the molecular pathogenesis of DM.

Our results showed significantly decreased Alu methylation in all of the samples in the increased HbA1c group during case follow‐up. We divided the samples into two subgroups based on the changes in HbA1c levels during the case follow‐up: those with decreased or equal HbA1c levels (good control) and those with increased HbA1c levels (bad control). Several studies have demonstrated that elevated HbA1c levels and hyperglycemia are crucial factors in the progression of type 2 DM,^52^, ^53^, ^54^ which explains why increased hyperglycemia is activated in response to various cellular stresses, including oxidative stress, inflammation, and DNA damage.^55^, ^56^, ^57^

The association between Alu methylation and increased HbA1c levels in type 2 DM patients might be linked to inflammatory disorders. Conversely, type 2 DM was also associated with elevated inflammatory markers.^58^ Several inflammatory markers have been identified, including serum uric acid, the uric acid/HDL cholesterol ratio, omentin, neuregulin, serum vitamin D, and hemogram‐based inflammatory markers like the platelet/lymphocyte ratio.^59^, ^60^, ^61^, ^62^, ^63^, ^64^ Thus, the increased HbA1c levels might correlate with inflammatory markers in type 2 DM patients. As a result, our findings suggest that managing diabetes, along with acknowledging the connection between heightened hyperglycemia and inflammatory complications in special populations such as elderly patients with type 2 DM and heart conditions, necessitates specific attention.^65^, ^66^

Accumulating evidence suggests that oxidative stress can directly damage DNA, leading to the formation of DNA adducts and strand breaks.^67^, ^68^ The presence of DNA damage triggers p53 activation as a protective response.^69^, ^70^ Moreover, p53 can directly induce tyrosine phosphorylation of insulin receptor substrate 1 (IRS1), and this effect has been found to inhibit insulin signaling and hinder glucose uptake. Consequently, increased DNA damage‐inducing p53 activity can lead to a decrease in IRS1 expression, alterations in IRS1 phosphorylation, and disruption of downstream insulin signaling pathways, ultimately contributing to insulin resistance.^71^, ^72^, ^73^, ^74^ Therefore, prolonged hyperglycemia causes an imbalance in oxidative production and suppression, which induces p53 and contributes to impaired insulin signaling, compromised glucose metabolism, and the pathogenesis of type 2 DM.^75^, ^76^

Our data suggest that decreased Alu methylation was more strongly associated with the poorly controlled blood glucose group, indicating a possible link between Alu hypomethylation and inadequate hyperglycemia management in type 2 DM. Importantly, previous studies have demonstrated that Alu methylation plays a role in preventing the accumulation of endogenous DNA damage.^30^ Therefore, Alu hypomethylation, which is associated with hyperglycemia, might induce DNA damage and potentially contribute to insulin resistance, reduced glucose uptake, elevated blood sugar levels, high HbA1c levels, and the development of type 2 DM.

Furthermore, we observed an inverse relationship between ΔAlu hypomethylation and ΔHbA1c in both normal control subjects and type 2 DM patients. These findings indicate that changes in blood glucose levels corresponded to changes in Alu hypomethylation, suggesting that decreased Alu methylation is associated with HbA1c levels. These findings are similar to those of HbA1c, which is an effective biomarker.^77^ The amount of Alu methylation is inversely associated with the degree of aging, and the degree of aging phenotype is directly associated with the severity of type 2 DM. Thus, Alu methylation levels could provide an overall picture of the average disease severity over a long period when detecting type 2 DM. In practical applications, it might be necessary to increase the sample size to generate a standardized graph demonstrating the decrease in Alu methylation in each age group, comparing individuals with diabetes to those without diabetes. Consequently, Alu methylation could potentially serve as a highly specific and novel biomarker for more accurate prediction and follow‐up of type 2 DM patients.

## 5 CONCLUSIONS

Our results not only showed significant Alu hypomethylation in type 2 DM but also demonstrated a decrease in Alu hypomethylation levels during the follow‐up of type 2 DM patients, particularly those who had increased HbA1c levels. This suggests that Alu hypomethylation might contribute to DNA damage and the subsequent decline in cellular function in type 2 DM. Therefore, Alu methylation could serve as a promising biomarker for monitoring type 2 DM and might facilitate the development of improved therapeutic approaches for prevention and treatment in the future.

## AUTHOR CONTRIBUTIONS

JT isolated DNA from plasma samples and processed the data. JT performed the Bisulfite DNA modification. JT and AM discuss the results part. JT and AM wrote the first draft of the manuscript and approved the final manuscript.

## FUNDING INFORMATION

This was financially supported by the Office of the Permanent Secretary, Ministry of Higher Education, Science, Research and Innovation Grant No. RGNS 63‐213, and the National Science and Technology Development Agency, Thailand [Research Chair Grant, P‐19‐50189 to A.M.].

## CONFLICT OF INTEREST STATEMENT

All the authors have no relationships or conflict of interest to declare.

## Supporting information


Figure S1

## Data Availability

The data that support the findings of this study are available from the corresponding author upon reasonable request.

## References

[1] Sun H , Saeedi P , Karuranga S , et al. IDF diabetes atlas: global, regional and country‐level diabetes prevalence estimates for 2021 and projections for 2045. Diabetes Res Clin Pract. 2022;183:109119. 34879977 10.1016/j.diabres.2021.109119 PMC11057359

[2] Guariguata L , Whiting DR , Hambleton I , Beagley J , Linnenkamp U , Shaw JE . Global estimates of diabetes prevalence for 2013 and projections for 2035. Diabetes Res Clin Pract. 2014;103(2):137‐149. 24630390 10.1016/j.diabres.2013.11.002

[3] Kovesdy C , Schmedt N , Folkerts K , et al. Predictors of cardio‐kidney complications and treatment failure in patients with chronic kidney disease and type 2 diabetes treated with SGLT2 inhibitors. BMC Med. 2022;20(1):1‐15. 35000594 10.1186/s12916-021-02191-2 PMC8744296

[4] Lee J , Yun J‐S , Ko S‐H . Advanced glycation end products and their effect on vascular complications in type 2 diabetes mellitus. Nutrients. 2022;14(15):3086. 35956261 10.3390/nu14153086 PMC9370094

[5] Zhou J , Zhang G , Chang C , et al. Metformin versus sulphonylureas for new onset atrial fibrillation and stroke in type 2 diabetes mellitus: a population‐based study. Acta Diabetol. 2022;59(5):697‐709. 35112189 10.1007/s00592-021-01841-4

[6] Ma Z‐Y , Wu Y‐Y , Cui H‐Y‐L , Yao G‐Y , Bian H . Factors influencing post‐stroke cognitive impairment in patients with type 2 diabetes mellitus. Clin Interv Aging. 2022;17:653‐664. 35520948 10.2147/CIA.S355242 PMC9063799

[7] Chai K‐C , Chen W‐M , Chen M , Shia B‐C , Wu S‐Y . Association between preexisting sarcopenia and stroke in patients with type 2 diabetes mellitus. J Nutr Health Aging. 2022;26:1‐9.3625958210.1007/s12603-022-1846-0

[8] Yao X , Pei X , Fan S , Yang X , Yang Y , Li Z . Relationship between renal and liver function with diabetic retinopathy in patients with type 2 diabetes mellitus: a study based on cross‐sectional data. Sci Rep. 2022;12(1):1‐9. 35672376 10.1038/s41598-022-13164-7 PMC9174192

[9] Raja SA , Chong VH , Rahman NA , Shakir LM , Knights J . Prevalence and associated factors of diabetic retinopathy among type 2 diabetes mellitus patients in Brunei Darussalam: a cross‐sectional study. Korean J Ophthalmol. 2022;36(1):26‐35. 34743489 10.3341/kjo.2021.0040 PMC8850000

[10] Eid M , Mounir A , El Etriby S , Al Taher A , Ezzat MA . Diabetic retinopathy as a predictor of angiographic coronary atherosclerosis severity in patients with type 2 diabetes mellitus. Diabetes, Metab Syndr Obes. 2022;15:1485‐1494. 35591908 10.2147/DMSO.S363406 PMC9113458

[11] Elhefnawy ME , Ghadzi SMS , Noor HS . Predictors associated with type 2 diabetes mellitus complications over time: a literature review. J Vasc Dis. 2022;1(1):13‐23.

[12] Yen H‐Y , Lee S‐C , Lin C‐F , Lee T‐I , Yamaguchi Y , Lee P‐H . Complications and comorbidities as influencing factors of health outcomes in older adults with type 2 diabetes mellitus. Collegian. 2022;30:230‐235.

[13] Beck RW , Bergenstal RM , Cheng P , et al. The relationships between time in range, hyperglycemia metrics, and HbA1c. J Diabetes Sci Technol. 2019;13(4):614‐626. 30636519 10.1177/1932296818822496 PMC6610606

[14] Davies MJ , Aroda VR , Collins BS , et al. Management of hyperglycemia in type 2 diabetes, 2022. A consensus report by the American Diabetes Association (ADA) and the European Association for the Study of diabetes (EASD). Diabetes Care. 2022;45(11):2753‐2786. 36148880 10.2337/dci22-0034 PMC10008140

[15] DasNandy A , Virge R , Hegde HV , Chattopadhyay D . A review of patent literature on the regulation of glucose metabolism by six phytocompounds in the management of diabetes mellitus and its complications. J Integr Med. 2023;21:226‐235. 36932029 10.1016/j.joim.2023.02.003

[16] Chehadeh SE , Sayed NS , Abdelsamad HS , et al. Genetic variants and their associations to type 2 diabetes mellitus complications in The United Arab Emirates. Front Endocrinol. 2021;12:12. 10.3389/fendo.2021.751885 PMC8772337 35069435

[17] DeForest N , Majithia AR . Genetics of type 2 diabetes: implications from large‐scale studies. Curr Diab Rep. 2022;1‐9:227‐235. 10.1007/s11892-022-01462-3 PMC9072491 35305202

[18] Curtis D . Analysis of rare coding variants in 200,000 exome‐sequenced subjects reveals novel genetic risk factors for type 2 diabetes. Diabetes Metab Res Rev. 2022;38(1):e3482. 34216101 10.1002/dmrr.3482

[19] Laakso M , Fernandes SL . Genetics of type 2 diabetes: past, present, and future. Nutrients. 2022;14(15):3201. 35956377 10.3390/nu14153201 PMC9370092

[20] Fraszczyk E , Spijkerman AM , Zhang Y , et al. Epigenome‐wide association study of incident type 2 diabetes: a meta‐analysis of five prospective European cohorts. Diabetologia. 2022;65(5):763‐776. 35169870 10.1007/s00125-022-05652-2 PMC8960572

[21] Rosik J , Szostak B , Machaj F , Pawlik A . The role of genetics and epigenetics in the pathogenesis of gestational diabetes mellitus. Ann Hum Genet. 2020;84(2):114‐124. 31571208 10.1111/ahg.12356

[22] Raciti GA , Desiderio A , Longo M , et al. DNA methylation and type 2 diabetes: novel biomarkers for risk assessment? Int J Mol Sci. 2021;22(21):11652. 34769081 10.3390/ijms222111652 PMC8584054

[23] Sae‐Lee C , Biasi JD , Robinson N , et al. DNA methylation patterns of LINE‐1 and Alu for pre‐symptomatic dementia in type 2 diabetes. PloS ONE. 2020;15(6):e0234578. 32525932 10.1371/journal.pone.0234578 PMC7289438

[24] Ahmed SAH , Ansari SA , Mensah‐Brown EP , Emerald BS . The role of DNA methylation in the pathogenesis of type 2 diabetes mellitus. Clin Epigenetics. 2020;12(1):1‐23. 10.1186/s13148-020-00896-4 PMC7353744 32653024

[25] Wang X , Yang J , Qiu X , Wen Q , Liu M , Chen Q . Blood DNA methylation and type 2 diabetes mellitus: a protocol for systematic review and meta‐analysis. Medicine. 2020;99(23):e20530. 32502009 10.1097/MD.0000000000020530 PMC7306281

[26] Tsai H‐H , Shen C‐Y , Ho C‐C , et al. Interaction between a diabetes‐related methylation site (TXNIP cg19693031) and variant (GLUT1 rs841853) on fasting blood glucose levels among non‐diabetics. J Transl Med. 2022;20(1):1‐9. 35164795 10.1186/s12967-022-03269-y PMC8842527

[27] Thongsroy J , Mutirangura A . The association between Alu hypomethylation and the severity of hypertension. PloS ONE. 2022;17(7):e0270004. 35802708 10.1371/journal.pone.0270004 PMC9269909

[28] Zhang J , Sheng H , Hu C , et al. Effects of DNA methylation on gene expression and phenotypic traits in cattle: a review. Int J Mol Sci. 2023;24(15):11882. 37569258 10.3390/ijms241511882 PMC10419045

[29] Petryk N , Bultmann S , Bartke T , Defossez P‐A . Staying true to yourself: mechanisms of DNA methylation maintenance in mammals. Nucleic Acids Res. 2021;49(6):3020‐3032. 33300031 10.1093/nar/gkaa1154 PMC8034647

[30] Patchsung M , Settayanon S , Pongpanich M , Mutirangura D , Jintarith P , Mutirangura A . Alu siRNA to increase Alu element methylation and prevent DNA damage. Epigenomics. 2018;10(2):175‐185. 29336607 10.2217/epi-2017-0096

[31] Pornthanakasem W , Kongruttanachok N , Phuangphairoj C , et al. LINE‐1 methylation status of endogenous DNA double‐strand breaks. Nucleic Acids Res. 2008;36(11):3667‐3675. 18474527 10.1093/nar/gkn261 PMC2441779

[32] Thongsroy J , Matangkasombut O , Thongnak A , Rattanatanyong P , Jirawatnotai S , Mutirangura A . Replication‐independent endogenous DNA double‐strand breaks in Saccharomyces cerevisiae model. PloS One. 2013;8(8):e72706. 23977341 10.1371/journal.pone.0072706 PMC3747138

[33] Thongsroy J , Patchsung M , Pongpanich M , Settayanon S , Mutirangura A . Reduction in replication‐independent endogenous DNA double‐strand breaks promotes genomic instability during chronological aging in yeast. FASEB J. 2018;32(11):6252‐6260. 10.1096/fj.201800218RR 29812972

[34] Yasom S , Watcharanurak P , Bhummaphan N , et al. The roles of HMGB1‐produced DNA gaps in DNA protection and aging biomarker reversal. FASEB BioAdv. 2022;4(6):408‐434. 35664831 10.1096/fba.2021-00131 PMC9164245

[35] Mutirangura A . A Hypothesis to Explain how the DNA of Elderly People Is Prone to Damage: Genome‐Wide Hypomethylation Drives Genomic Instability in the Elderly by Reducing Youth‐Associated Gnome‐Stabilizing DNA Gaps. In: Meccariello R , ed. Epigenetics. IntechOpen; 2018.

[36] Çalışkan Z , Mutlu T , Güven M , et al. SIRT6 expression and oxidative DNA damage in individuals with prediabetes and type 2 diabetes mellitus. Gene. 2018;642:542‐548. 29197589 10.1016/j.gene.2017.11.071

[37] Oguntibeju OO . Type 2 diabetes mellitus, oxidative stress and inflammation: examining the links. Int J Physiol Pathophysiol Pharmacol. 2019;11(3):45‐63. 31333808 PMC6628012

[38] Binici D , Karaman A , Coşkun M , Oğlu AU , Uçar F . Genomic damage in patients with type‐2 diabetes mellitus. Genet Couns. 2013;24(2):149‐156. 24032284

[39] López‐Otín C , Blasco MA , Partridge L , Serrano M , Kroemer G . The hallmarks of aging. Cell. 2013;153(6):1194‐1217. 23746838 10.1016/j.cell.2013.05.039 PMC3836174

[40] Narasimhan A , Flores RR , Robbins PD , Niedernhofer LJ . Role of cellular senescence in type II diabetes. Endocrinology. 2021;162(10):bqab136. 34363464 10.1210/endocr/bqab136 PMC8386762

[41] Pippitt K , Li M , Gurgle HE . Diabetes mellitus: screening and diagnosis. Am Fam Physician. 2016;93(2):103‐109. 26926406

[42] Valadan M , Bahramnezhad Z , Golshahi F , Feizabad E . The role of first‐trimester HbA1c in the early detection of gestational diabetes. BMC Pregnancy Childbirth. 2022;22(1):71. 35086491 10.1186/s12884-021-04330-2 PMC8793236

[43] Association AD . Standards of medical care in diabetes—2019 abridged for primary care providers. Clin Diabetes. 2019;37(1):11‐34. 30705493 10.2337/cd18-0105 PMC6336119

[44] Inzucchi SE , Bergenstal RM , Buse JB , et al. Management of hyperglycemia in type 2 diabetes: a patient‐centered approach: position statement of the American Diabetes Association (ADA) and the European Association for the Study of diabetes (EASD). Diabetes Care. 2012;35(6):1364‐1379. 22517736 10.2337/dc12-0413 PMC3357214

[45] Sherwani SI , Khan HA , Ekhzaimy A , Masood A , Sakharkar MK . Significance of HbA1c test in diagnosis and prognosis of diabetic patients. Biomark Insights. 2016;11:95‐104. 27398023 10.4137/BMI.S38440 PMC4933534

[46] Berhe KK , Gebru HB , Kahsay HB . Effect of motivational interviewing intervention on HgbA1C and depression in people with type 2 diabetes mellitus (systematic review and meta‐analysis). PloS ONE. 2020;15(10):e0240839. 33095794 10.1371/journal.pone.0240839 PMC7584232

[47] Yooyongsatit S , Ruchusatsawat K , Noppakun N , Hirankarn N , Mutirangura A , Wongpiyabovorn J . Patterns and functional roles of LINE‐1 and Alu methylation in the keratinocyte from patients with psoriasis vulgaris. J Hum Genet. 2015;60(7):349‐355. 25833468 10.1038/jhg.2015.33

[48] Sirivanichsuntorn P , Keelawat S , Danuthai K , Mutirangura A , Subbalekha K , Kitkumthorn N . LINE‐1 and Alu hypomethylation in mucoepidermoid carcinoma. BMC Clin Pathol. 2013;13(1):1‐11. 23510117 10.1186/1472-6890-13-10 PMC3610265

[49] Kitkumthorn N , Keelawat S , Rattanatanyong P , Mutirangura A . LINE‐1 and Alu methylation patterns in lymph node metastases of head and neck cancers. Asian Pac J Cancer Prev. 2012;13(9):4469‐4475. 23167363 10.7314/apjcp.2012.13.9.4469

[50] Thongsroy J , Patchsung M , Mutirangura A . The association between Alu hypomethylation and severity of type 2 diabetes mellitus. Clin Epigenetics. 2017;9(1):93. 28883893 10.1186/s13148-017-0395-6 PMC5580285

[51] Jintaridth P , Tungtrongchitr R , Preutthipan S , Mutirangura A . Hypomethylation of Alu elements in post‐menopausal women with osteoporosis. PloS One. 2013;8(8):e70386. 23990903 10.1371/journal.pone.0070386 PMC3749148

[52] Takahara M , Mita T , Katakami N , et al. Three‐year glycaemic control and management in patients with type 2 diabetes initiating second‐line treatment in Japan: a prospective observational study, J‐DISCOVER. Diabetes Ther. 2022;13(2):251‐264. 34962628 10.1007/s13300-021-01192-x PMC8873328

[53] Nalysnyk L , Hernandez‐Medina M , Krishnarajah G . Glycaemic variability and complications in patients with diabetes mellitus: evidence from a systematic review of the literature. Diabetes Obes Metab. 2010;12(4):288‐298. 20380649 10.1111/j.1463-1326.2009.01160.x

[54] Han F , Shi X‐l , Pan J‐j , et al. Elevated serum HbA1c level, rather than previous history of diabetes, predicts the disease severity and clinical outcomes of acute pancreatitis. BMJ Open Diabetes Res Care. 2023;11(1):e003070. 10.1136/bmjdrc-2022-003070 PMC9906177 36746527

[55] Giri B , Dey S , Das T , Sarkar M , Banerjee J , Dash SK . Chronic hyperglycemia mediated physiological alteration and metabolic distortion leads to organ dysfunction, infection, cancer progression and other pathophysiological consequences: an update on glucose toxicity. Biomed Pharmacother. 2018;107:306‐328. 30098549 10.1016/j.biopha.2018.07.157

[56] Gheitasi I , Savari F , Akbari G , Mohammadi J , Fallahzadeh AR , Sadeghi H . Molecular mechanisms of hawthorn extracts in multiple organs disorders in underlying of diabetes: a review. Int J Endocrinol. 2022;2022:1‐14. 10.1155/2022/2002768 PMC9197671 35711333

[57] Yang X , Zhang R , Jin T , et al. Stress hyperglycemia is independently associated with persistent organ failure in acute pancreatitis. Dig Dis Sci. 2022;67(5):1879‐1889. 33939149 10.1007/s10620-021-06982-8 PMC9142444

[58] Cakir L , Aktas G , Enginyurt O , Cakir SA . Mean platelet volume increases in type 2 diabetes mellitus independent of HbA1c level. Acta Medica Mediterranea. 2014;30(2):425‐428.

[59] Atak BM , Duman TT , Kocak MZ , Savli H . Serum uric acid level is associated with type 2 diabetes mellitus and diabetic regulation. Exp Biomed Res. 2018;1(4):135‐139.

[60] Aktas G , Kocak MZ , Bilgin S , Atak BM , Duman TT , Kurtkulagi O . Uric acid to HDL cholesterol ratio is a strong predictor of diabetic control in men with type 2 diabetes mellitus. Aging Male. 2020;23(5):1098‐1102. 31615320 10.1080/13685538.2019.1678126

[61] Aktas G , Alçelik A , Ozlu T , et al. Association between omentin levels and insulin resistance in pregnancy. Exp Clin Endocrinol Diabetes. 2014;122(3):163‐166. 24643693 10.1055/s-0034-1370917

[62] Koçak MZ , Aktaş G , Erkuş E , et al. Neuregulin‐4 is associated with plasma glucose and increased risk of type 2 diabetes mellitus. Swiss Med Wkly. 2019;149:w20139. 31656034 10.4414/smw.2019.20139

[63] Erkus E , Aktas G , Kocak MZ , Duman TT , Atak BM , Savli H . Diabetic regulation of subjects with type 2 diabetes mellitus is associated with serum vitamin D levels. Rev Assoc Med Bras. 2019;65:51‐55. 30758420 10.1590/1806-9282.65.1.51

[64] Atak B , Aktas G , Duman TT , Erkus E , Kocak MZ , Savli H . Diabetes control could through platelet‐to‐lymphocyte ratio in hemograms. Rev Assoc Med Bras. 2019;65:38‐42. 30758418 10.1590/1806-9282.65.1.38

[65] Aktas G , Atak Tel BM , Tel R , Balci B . Treatment of type 2 diabetes patients with heart conditions. Expert Rev Endocrinol & Metab. 2023;18(3):255‐265. 37078758 10.1080/17446651.2023.2204941

[66] Tel BMA , Bilgin S , Kurtkulagi O , et al. Frailty in diabetic subjects during COVID‐19 and its association with HbA1c, mean platelet volume and monocyte/lymphocyte ratio. Clin Diabetol. 2022;11(2):119‐126.

[67] Cadet J , Wagner JR . Oxidatively generated base damage to cellular DNA by hydroxyl radical and one‐electron oxidants: similarities and differences. Arch Biochem Biophys. 2014;557:47‐54. 24820329 10.1016/j.abb.2014.05.001

[68] De Bont R , Van Larebeke N . Endogenous DNA damage in humans: a review of quantitative data. Mutagenesis. 2004;19(3):169‐185. 15123782 10.1093/mutage/geh025

[69] Williams AB , Schumacher B . p53 in the DNA‐damage‐repair process. Cold Spring Harb Perspect Med. 2016;6(5):a026070. 27048304 10.1101/cshperspect.a026070 PMC4852800

[70] Wang YH , Sheetz MP . Transcription‐independent functions of p53 in DNA repair pathway selection. Bioessays. 2023;45(1):2200122. 10.1002/bies.202200122 36404121

[71] Xi G , Shen X , Wai C , White MF , Clemmons DR . Hyperglycemia induces vascular smooth muscle cell dedifferentiation by suppressing insulin receptor substrate‐1–mediated p53/KLF4 complex stabilization. J Biol Chem. 2019;294(7):2407‐2421. 30578299 10.1074/jbc.RA118.005398 PMC6378959

[72] Peng P , Ma C , Wan S , et al. Inhibition of p53 relieves insulin resistance in fetal growth restriction mice with catch‐up growth via activating IGFBP3/IGF‐1/IRS‐1/Akt signaling pathway. J Nanosci Nanotechnol. 2018;18(6):3925‐3935. 29442728 10.1166/jnn.2018.15036

[73] Machado‐Neto JA , Fenerich BA , Rodrigues Alves APN , et al. Insulin substrate receptor (IRS) proteins in normal and malignant hematopoiesis. Clinics. 2018;73:e566s. 30328953 10.6061/clinics/2018/e566s PMC6169455

[74] Cui D , Qu R , Liu D , Xiong X , Liang T , Zhao Y . The cross talk between p53 and mTOR pathways in response to physiological and genotoxic stresses. Front Cell Dev Biol. 2021;9:3320. 10.3389/fcell.2021.775507 PMC8638743 34869377

[75] Newsholme P , Cruzat VF , Keane KN , Carlessi R , de Bittencourt Jr PIH . Molecular mechanisms of ROS production and oxidative stress in diabetes. Biochem J. 2016;473(24):4527‐4550. 27941030 10.1042/BCJ20160503C

[76] Strycharz J , Drzewoski J , Szemraj J , Sliwinska A . Is p53 involved in tissue‐specific insulin resistance formation? Oxid Med Cell Longev. 2017;2017:1‐23. 10.1155/2017/9270549 PMC5282448 28194257

[77] Matsushita Y , Yokoyama T , Takeda N , et al. A comparison in the ability to detect diabetic retinopathy between fasting plasma glucose and HbA1c levels in a longitudinal study. Endocrinol, Diabetes & Metab. 2021;4(1):e00196. 33532623 10.1002/edm2.196 PMC7831218

