# Supplementary figures and images for: Decreased Alu methylation in type 2 diabetes mellitus patients increases HbA1c levels
Source: J Clin Lab Anal. 2023 Sep 24;37(17-18):e24966. doi: 10.1002/jcla.24966 (PMC10623537; doi:10.1002/jcla.24966)

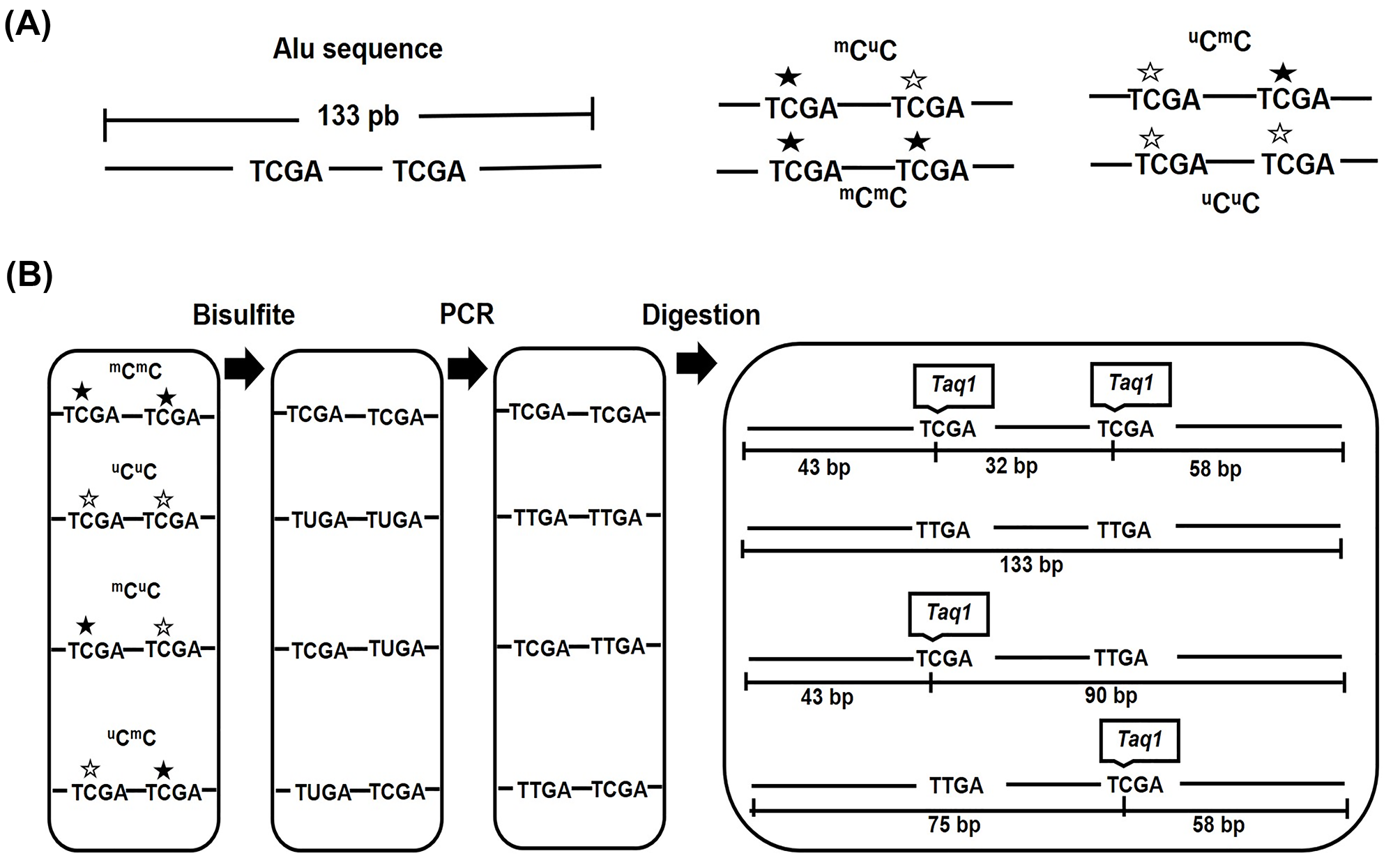

Supplement: Supplementary file 1 — Figure S1 [file JCLA-37-e24966-s001.tif]
